# The Effect of the Charlson Comorbidity Index On In-Hospital Complications, Hospital Length of Stay, Mortality, and Readmissions Among Patients Hospitalized for Acute Stroke

**DOI:** 10.7759/cureus.60112

**Published:** 2024-05-11

**Authors:** Niaz Shaikh, Asna Mohammed, Mahdis Seddiq, Samreen Kidwai, Dania Shahzad, Mariem M Mahmoud

**Affiliations:** 1 Internal Medicine, Rashid Hospital, Dubai, ARE; 2 Internal Medicine, Hackensack University Medical Center, Hackensack, USA; 3 Emergency Medicine, Rashid Hospital, Dubai, ARE

**Keywords:** readmission, mortality, hospital complications, charlson comorbidity index, cerebrovascular accident (stroke)

## Abstract

Aim

A notable number of people who develop stroke have comorbid medical conditions. The aim of this study is to evaluate the use of the Charlson Comorbidity Index (CCI) to predict in-hospital complications, mortality, length of stay, and readmission rates in stroke patients.

Method

It is a retrospective study that analyzed patients who were admitted for stroke in a six-month time duration. Stroke was classified into ischemic, hemorrhagic, or undetermined; hospital complications were classified into medical or neurological. Data regarding comorbidities, complications, length of stay, mortality, and readmissions were documented. Comorbidities were then classified by the CCI and split into four categories: zero, mild (1-2), moderate (3-4) and severe (5+). The data was analyzed using SPSS (IBM, Inc., Armonk, US).

Results

Four hundred and seventy-three adults aged above 18 were hospitalized for acute stroke. There was no correlation between the severity of the CCI score and mortality. Patients with ischemic stroke had a higher CCI correlated with readmission rate (p=0.026) and hospital complications (p=0.054). The two groups with the highest intensive care unit admission rate were mild, followed by the severe group (p=0.001). Our study also revealed that the patients with severe CCI scores had an increased readmission rate (p=0.001).

Conclusion

There is a correlation between a high CCI score and readmission rate, as well as CCI score with hospital complications in ischemic stroke. Further prospective studies of a longer duration can be undertaken to find further associations with the potential for this score to be used as a predictor in patients hospitalized for stroke.

## Introduction

Cerebrovascular diseases are important causes of global morbidity and mortality. The Centers for Disease Control and Prevention (CDC) reported that in the year 2021, one in every six deaths from cardiovascular disease was due to stroke [1]. Stroke remains the second-leading cause of death and the third-leading cause of death and disability combined in the world, with the bulk of the global stroke burden residing in lower-income and lower-middle-income countries [2].‌ In the Middle East and North Africa alone, the rise in stroke incidences is a major health concern, with data predicting stroke mortality will double by the year 2030. According to the Health Authority of Abu Dhabi (HAAD), in 2014, 37% of mortality in the United Arab Emirates was attributed to circulatory disorders (including stroke and ischemic heart disease) [3]. The stroke incidence rates in the Middle East have increased dramatically over the past decade, exceeding the level seen in some high-income countries [4].

Patients hospitalized with acute cerebrovascular accidents can develop various medical and neurological complications. Commonly reported complications include brain edema, infections, deep vein thrombosis, and depression [1]. Medical comorbidities such as hypertension, diabetes, congestive heart disease, and atrial fibrillation are established risk factors for the development of stroke, but they may also result in increased risk of complications, length of in-hospital stay, mortality rate, and result in overall poor prognosis [5]. Up to 90% of stroke patients have one or more comorbid conditions, and almost a quarter have ≥5 chronic medical conditions [3]. The incidence of stroke is higher in the elderly population as many of them have a high pre-existing comorbid burden. This can have an impact on the outcome, including mortality, as well as long-term functional limitations and cognitive impairment.

The Charlson Comorbidity Index (CCI) is the most commonly used case-mix adjustment method in health outcome studies that use administrative data. Using a population of general medical inpatients at one hospital, Charlson identified 17 comorbidities that were associated with one-year mortality and assigned weights to these conditions that, when summed, created an index predicting mortality [6]. Higher scores have been linked with longer lengths of hospital stay and worse outcomes on discharge [3]. Furthermore, a systematic review by Rashid et al. found that a high comorbid burden, as defined by CCI, is associated with a significant increase in the risk of mortality in patients with underlying coronary artery disease, heart failure, and cerebrovascular accidents [7]. Studies have also demonstrated that older patients who were hospitalized for acute stroke had worse clinical outcomes when their CCI score was higher, though the association was also found to be dependent on stroke severity and premorbid disability in one study [8-11].

The present study evaluates the comorbidities among stroke patients as measured by the Charlson Comorbidity Index and its effect on in-hospital complications, length of hospital stay, mortality, and readmissions within four weeks following discharge. This is one of the first studies based on the Charlson Comorbidity Index taking place in the United Arab Emirates (UAE).

## Materials and methods

Study design and population

This is a retrospective study of all the patients admitted for acute stroke between 1 July 2019 and 31 December 2019 to Rashid Hospital, a tertiary specialized hospital in Dubai, United Arab Emirates. The data was collected through the Dubai Health Authority's electronic medical records registry, Salama.

The criteria for stroke applied in this study included intracerebral hemorrhage, cerebral infarction and undetermined stroke (not specified as hemorrhage or infarction, i.e. unknown mechanism). As per the International Classification of Diseases (ICD), version 10, this comprised the ICD codes I60 to I68. Patients aged 18 and older with a discharge diagnosis of stroke with the mentioned ICD codes were included in this research.

Excluded from this study were patients less than 18 years of age, cases with incomplete documentation of comorbidity and complications, any patient discharged against medical advice, as well as patients with traumatic intracranial hemorrhage.

Comorbidities were classified via the Charlson Comorbidity Index and were inputted into an MS Excel (Microsoft Corp., Redmond, WA) sheet for CCI score calculation. The data regarding the presence of documented complications, length of stay, mortality, and readmission after four weeks from discharge date, were also added into an Excel format sheet and reviewed via SPSS 24 (IBM, Inc., Armonk, NY) analysis.

Covariates

Demographic details included the patient's age, sex, and nationality. Clinical data included the type of stroke, in addition to the length of hospital stay, admission into the ICU, hospital complications, readmission within four weeks, and in-hospital mortality. The hospital complications were divided into medical (including infection, pulmonary embolism, and electrolyte disturbances) and neurological (seizures, headache, and dizziness).

The Charlson Comorbidity Index is a weighted scoring system that is based on the classification of comorbidities, which provides a validated means to predict the risk of death from comorbid disease. For all the patients, the score was calculated based on 17 comorbid conditions included in the index that were present prior to the stroke occurrence, namely, congestive heart failure (one point), myocardial infarction (one point), cerebrovascular disease (one point), chronic pulmonary disease (one point), paraplegia (two points), dementia (one point), diabetes without complications (one point), diabetes with complications (two points), cancer (two points), metastatic cancer (six points), mild liver disease (one point), moderate or severe liver disease (three points), peptic ulcer disease (one point), peripheral vascular disease (one point), rheumatologic disease (one point), renal disease (two points), and human immunodeficiency virus (HIV)/acquired immune deficiency syndrome (AIDS) (six points).

In addition, we also collected data on other factors that are not part of the CCI calculation, such as hypertension, dyslipidemia, atrial fibrillation, obesity, and smoking status.

Statistical analyses

Numerical data is presented as mean ± standard deviation for bell-shaped data, median and range for skewed data, and categorical data is presented as count and percent. The CCI score was categorized into four categories: zero (0), mild (1-2), moderate (3-4) and severe (5+). The chi-squared test was used to compare categorical responses (mortality, hospital complications, readmission within four weeks, and intensive care unit [ICU] admission) between the groups of CCI and between the groups of stroke types. Kruskal-Wallis test was used to compare the length of stay between the groups of CCI and between the groups of stoke type. SPSS 24 was used for data analysis. P-value <0.05 indicates statistical significance. 

## Results

Characteristics

Between July 1st, 2019, and December 31st, 2019, 473 adults aged above 18 were hospitalized for acute stroke. Two hundred and eighteen (46.1%) were less than 50 years old; 74.4% were male, and the predominant ethnicity was South Asian (56.7%). Among types of stroke, 63% were ischemic, 33.4% were hemorrhagic, and 3.6% were undetermined with an unknown mechanism of stroke.

The mean CCI score was 2.11 (SD±2.043), and the median was 1 with a range of 0-11. The CCI score was split into four categories: zero, mild (1-2), moderate (3-4) and severe (5+) [12]. Zero CCI score was observed in 21.1%, 45.5% were in the mild category, 19.9% were in the moderate and 13.5% were in the severe category. Thus, 66.7% of the patients had zero or mild CCI scores, with the rest (33.3%) being moderate or severe (3+). Other patient characteristics are described in Table 1. 

**Table 1:** Sociodemographic and clinical characteristics of patients hospitalized for acute stroke CCI - Charlson Comorbidity Index; CVA - cerebrovascular accident; TIA - transient ischemic attack; COPD - chronic obstructive pulmonary disease; CKD - chronic kidney disease; ICU - intensive care unit

Characteristics	Total (n=473)	%
Age		
<50	218	46.10%
50-59	109	23%
60-69	73	15.40%
70-79	52	11%
>80	21	4.40%
Gender		
Male	352	74.40%
Female	121	25.60%
Ethnicity		
Southeast Asia	69	14.60%
East Asia	8	1.70%
South Asia	268	56.70%
Western Asia	75	15.90%
Central Asia	36	7.60%
Africa	8	1.70%
Europe	9	1.90%
Types of stroke		
Ischemic	296	63%
Hemorrhagic	158	33.40%
Undetermined	19	3.60%
Comorbidities according to CCI		
Congestive heart failure	23	4.90%
Myocardial infarction	36	7.60%
CVA or TIA	61	12.90%
COPD	0	0
Hemiplegia	6	1.30%
Dementia	12	2.50%
Uncomplicated diabetes (on medication)	100	21.10%
Diabetes with end organ damage	63	13.30%
Solid tumor localized	2	0.40%
Solid tumor metastatic	1	0.2 %
Liver disease (mild)	2	0.40%
Liver disease (moderate-severe)	1	0.20%
Peptic ulcer disease	7	1.50%
Peripheral vascular disease	5	1.10%
Connective tissue disease	3	0.60%
Moderate-severe CKD	17	3.60%
Leukemia	0	0
Lymphoma	1	0.2 %
AIDS	0	0
Other comorbidities		
Hypertension	331	70%
Dyslipidemia	149	31.50%
Atrial fibrillation	26	5.50%
Obesity	39	8.20%
Smoking	74	15.60%
Admitted to ICU	107	22.8%
Developed in-hospital complications	120	25.4%
Medical complications		17.80%
Neurological complications		3.40%
Both		4.20%
Mortality	38	8%
Readmission within four weeks	14	3%
Median length of stay	6 days	

Mortality, hospital complications, ICU admission, and readmission within four weeks

Infection was the most common medical complication, and urinary tract infection was the most frequently reported in 24 (5.1%) patients, followed by a chest infection that was found in 15 (3.2%) patients. Eighteen (3.8%) patients had more than one type of infection simultaneously. Table 2 describes the mortality, hospital complications, ICU admission, and readmissions within four weeks of discharge.

**Table 2:** Mortality, hospital complications, ICU admission, readmission within four weeks *Excluding those who passed away, n=435 CCI - Charlson Comorbidity Index; ICU - intensive care unit

Variables	All (n=473)	CCI				p-value
		Zero	Mild	Moderate	Severe	
Mortality, n (%)	38 (8)	9 (23.7)	17 (44.7)	6 (15.8)	6 (15.8)	0.889
Ischemic	16	4	5	4	3	0.8
Hemorrhagic	22	5	12	2	3	0.383
Undetermined	0	0	0	0	0	
Hospital complications, n (%)	120 (25.4)	28 (23.3)	47 (39.2)	25 (20.8)	20 (16.7)	0.391
Ischemic	55	10	16	14	15	0.054
Hemorrhagic	65	18	31	11	5	0.811
Undetermined	0	0	0	0	0	
ICU admission, n (%)	107 (22.8)	37 (34.6)	39 (36.4)	16 (15)	15 (14)	0.001
Ischemic	36	10	6	8	12	0.002
Hemorrhagic	71	27	33	8	3	0.093
Undetermined	0	0	0	0	0	
Readmission within four weeks, n (%)*	14 (3.2)	2 (14.3)	1 (7.1)	5 (35.7)	6 (42.9)	0.001
Ischemic	10	1	0	4	5	0.026
Hemorrhagic	4	1	1	1	1	0.426
Undetermined	0	0	0	0	0	

Length of stay 

The median length of stay across all groups was six days (range: 5-7 days) across all CCI subgroups (zero, mild, moderate, severe). In patients with undetermined stroke, the median length was two days regardless of CCI score (p=0.001). In patients with ischemic stroke, the median length of stay was between 5 to 5.5 days in all groups except in the severe one, which was 6.5 days (p<0.001). In patients with hemorrhagic stroke, the median length of stay was between 8-15 days across the various CCI groups, the highest of which was found in the mild CCI group (15 days), the lowest of which was in the severe group (eight days, p<0.001).

## Discussion

This study population consisted of those from the ages of 18 and above with a multi-ethnic background, a diversity not commonly found in other research articles of similar study design and objectives. We note that 46% of our patient population was less than 50 years old, which we hypothesize is related to the predominant South Asian ethnicity, a population known to develop cardiovascular and cerebrovascular complications at a comparatively younger age compared to other ethnicities [13]. While similar studies analyzed the association of CCI with mortality and length of hospital stay, we also wanted to highlight other important aspects, including relation to hospital complications, ICU admission, and readmissions [8,12]. 

Length of hospital stay, mortality, and complications

The length of stay showed no relation with the CCI subgroups. 

We did not find any relationship between CCI and mortality as well as with the hospital complication rates. When separating the data into the type of stroke, those in the severe CCI group of ischemic stroke had the highest rate of developing hospital complications, followed by the moderate and then the mild group. One explanation could be that those with ischemic stroke were in a higher age group than those with hemorrhagic stroke (median age 50-59 with ischemic stroke, <50 with hemorrhagic stroke, respectively). This may suggest that the CCI severity score may be used as a tool for assessing the risks of hospital complications for this category of patients. 

ICU admission rates

When analyzing ICU admission rates, the two groups with the highest ICU admission were CCI group with a zero score, followed by the severe CCI group with a significant p-value. The mild CCI group was reported to have a higher ICU admission rate despite having less comorbidity. This may be due to the reason that patients were not aware of their underlying comorbidities leading to a lower CCI score with ICU admission.

It is also interesting to point out that when analyzing the ICU admission with specific types of stroke, ischemic, hemorrhagic, and undetermined strokes, the hemorrhagic group showed no relation of the CCI with ICU admission. This may be due to the smaller sample size among the hemorrhagic group. However, the ischemic group had a high ICU admission among the severe CCI group, followed by the moderate and then mild groups. This data indicates a reasonable use of the CCI for ICU admission risk assessment for patients with ischemic stroke admission. Using CCI on such patients may allow physicians to categorize high-risk patients requiring closer monitoring. 

Readmission rates

The severity of the CCI score may be used as an indicator of the readmission rate for stroke patients. Our study revealed that the patients with severe CCI had an increased rate of readmission. Fourteen (3%) patients were readmitted within four weeks, and the majority of them (n=11) belonged to moderate and severe CCI groups. A similar study in Australia also found a link between 90-day readmission of stroke patients and a high CCI score [14]. Interestingly, patients with a previous history of cerebrovascular accident (CVA)or transient ischemic attack (TIA), which is included in the CCI score, had no increased risk in the development of hospital complications, increased rate of readmission, ICU admission, or mortality. 

There are many limitations of this study, including single-center data and retrospective collection of the data from the records. Patients may have been unaware of their medical comorbidities or may be unable to narrate because of the neurological deficit. There might have been coding issues that might have missed some of the medical comorbidities. Some of the patients remained hospitalized for social reasons, including repatriation. We take into account the possibility of a limitation regarding readmission rates, as some patients may have returned to their home country for continued care. Other studies around the world, including those performed in Italy and Australia, found a more significant association between CCI and mortality, specifically among elderly patients [8,9]. Studies in Saudi Arabia, Turkey, and China also note an increase in stroke mortality with aging [15-17]. Mortality rates among stroke patients have been shown to vary from 8 to 28% in the Middle East, though other regions report higher mortality rates up to 35% [4,16]. Our study had a relatively lower mortality rate of 8% compared to other studies, which may be explained by the younger age group of our population. Our study further provides information on this age group, highlighting more significant findings related to ICU admission, readmissions, and in-hospital complications.

Patients with higher CCI scores and admitted with ischemic strokes have increased ICU admissions and readmissions. Given this outcome, our study supports using CCI as a risk assessment in these particular patients. Clinicians may benefit from using the CCI score in patients who are aware of their underlying comorbidities, as this may aid them in categorizing patients who require greater monitoring due to their increased risk of developing poorer outcomes during their hospital stay. This could also be used by clinicians to help educate their patients on their readmission risk and emphasize the need to control their comorbidities better. In addition, CCI has also been shown to be predictive of long-term outcomes up to six months to one year following stroke. Goldstein et al. found that functional outcomes at the time of hospital discharge and one-year mortality rates are associated with the number and severity of comorbidities, as reflected by a modified version of the CCI [18]. Caballero et al. similarly found that those with a higher CCI score had increased odds of poor outcomes and death at six months [19]. This further supports the use of CCI in predicting both short-term and long-term outcomes in this patient population. 

Further studies may be performed to control for age, stroke severity, and pre-stroke functional status, all of which may potentially have a significant impact on the outcomes measured independent of the CCI score. Additionally, further studies may also be done to assess the various types of strokes, with a larger sample size as a prospective study. The CCI score may also be studied prospectively as a risk indicator for future strokes in a patient previously hospitalized for stroke. Such studies can help emphasize the importance of controlling comorbidities among the population as data reveals that comorbidity does not only lead to stroke but also increases the risk of ICU admission, hospital complication and readmission, and longer in-hospital stay.

## Conclusions

Given the global morbidity of stroke, it is important to find indices that may be used by physicians to closely monitor certain patients. Though the CCI score was not initially intended to be used for this purpose, results from our study revealed a significant correlation between stroke and readmission rates, in addition to ischemic stroke and hospital complications. As this was a retrospective study, using the CCI when admitting stroke patients and screening for the relevant comorbidity may allow physicians to recognize the high-risk groups and manage them accordingly.
